# Nano-scale charge trapping memory based on two-dimensional conjugated microporous polymer

**DOI:** 10.1038/s41598-023-44232-1

**Published:** 2023-11-01

**Authors:** Ayman Rezk, Md. Hasan Raza Ansari, Kayaramkodath Chandran Ranjeesh, Safa Gaber, Dayanand Kumar, Areej Merhi, Bilal R. Kaafarani, Mohamed Ben Hassine, Nazek El-Atab, Dinesh Shetty, Ammar Nayfeh

**Affiliations:** 1 Department of Electrical Engineering and Computer Science, Khalifa University, Abu Dhabi, 127788 UAE; 2 Smart, Advanced Memory Devices and Applications (SAMA) Laboratory, Electrical and Computer Engineering Program, Computer Electrical Mathematical Science and Engineering Division, King Abdullah University of Science and Technology (KAUST), 23955 Thuwal, Kingdom of Saudi Arabia; 3 Department of Chemistry, Khalifa University, PO Box 127788, Abu Dhabi, UAE; 4 Department of Chemistry, American University of Beirut, Beirut, 1107-2020 Lebanon; 5 Electron Microscopy Core Labs, King Abdullah University of Science and Technology (KAUST), 23955 Thuwal, Kingdom of Saudi Arabia; 6 Advanced Materials Chemistry Center (AMCC), Khalifa University, PO Box 127788, Abu Dhabi, UAE

**Keywords:** Nanoscience and technology, Nanoscale devices, Electronic devices

## Abstract

There is a growing interest in new semiconductor nanostructures for future high-density high-performance flexible electronic devices. Two-dimensional conjugated microporous polymers (2D-CMPs) are promising candidates because of their inherent optoelectronic properties. Here, we are reporting a novel donor–acceptor type 2D-CMP based on Pyrene and Isoindigo (**PI**) for a potential nano-scale charge-trapping memory application. We exfoliated the **PI** polymer into ~ 2.5 nm thick nanoparticles (NPs) and fabricated a Metal–Insulator–Semiconductor (MIS) device with **PI**–NPs embedded in the insulator. Conductive AFM (cAFM) is used to examine the confinement mechanism as well as the local charge injection process, where ultrathin high-κ alumina supplied the energy barrier for confining the charge carrier transport. We have achieved a reproducible on-and-off state and a wide memory window (ΔV) of 1.5 V at a relatively small reading current. The device displays a low operation voltage (V < 1 V), with good retention (10^4^ s), and endurance (10^3^ cycles). Furthermore, a theoretical analysis is developed to affirm the measured charge carriers’ transport and entrapment mechanisms through and within the fabricated MIS structures. The **PI**–NPs act as a nanoscale floating gate in the MIS-based memory with deep trapping sites for the charged carriers. Moreover, our results demonstrate that the synthesized 2D-CMP can be promising for future low-power high-density memory applications.

## Introduction

Organic electroactive memory materials are advantageous compared to their inorganic counterparts because they are cheaper, easier to fabricate, and can achieve flexibility, deformability, and more importantly, tunable electrical characteristics by using a molecular design approach. Recently, two-dimensional conjugated microporous polymers (2D-CMPs)^1–5^ are gaining significant attention in this field due to their extensive π-conjugation, excellent chemical and thermal stabilities, tunable layered porous structures, and functions. In fact, for over a decade, they have been utilized in successful commercial applications, such as anti-static bags, magnetic storage, capacitors, batteries, and electrostatic loudspeakers^6–8^.

Electroactive memory materials including CMPs exhibit both electrically volatile and non-volatile memory (NVM) characteristics^9,1, 10, 11^. The conjugated backbone of CMPs is the primary contributor to their electrical properties. When various donors and acceptors are incorporated into the CMPs, the memory properties can be significantly influenced, which can either establish trapping sites or create charge transfer (CT) conducting channels. Making CMPs intriguing as the volatility of the memory device is determined by the stability of charge trapping. However, because of the cross-linked configuration and non-solubility in most solvents, forming thin films from CMPs is a major obstacle to their incorporation in optoelectronic devices. The electrical properties of these devices can be characterized at the nanoscale using conductive atomic force microscopy (cAFM), hence providing a useful method for studying electronic transport and conduction in such structures without the need for thin film formation.

Nanoscale memory devices can utilize nanoparticles (NPs) as isolated charge trap sites. These devices reap the benefits of conventional NVM while improving the stability of the stored information^12–14^. The enhanced stability in charge storage is mainly associated with the quantum confinement and electrical isolation of the NPs, allowing for both higher power efficiency and density. One approach to achieve this is by embedding semiconductor or metallic NPs in a floating gate layer made of dielectric oxides. In this case, the charged carriers are injected from and to the active region through direct tunneling. The trapped charge carriers in the NPs dampen out the electric field through the dielectric and shift the device's threshold voltage approximately by the number of carriers trapped within each NP. The trapped charge can also be erased by applying a sufficient voltage bias.

Several studies^9,1, 10,11^ have explored the hybridization of nanoscale NVMs and organic thin film devices (OTFDs). The goal is to leverage the benefits of OTFDs, such as processability, low cost, and the ability to fabricate large-area structures on flexible substrates. Unlike traditional NVMs, which rely on more rigid solid-state structures, many flexible electronic devices require optimized nonvolatile data storage. However, previous approaches for charge trapping in the OTFD have used CPs as a continuous charge-trapping layer to replace the floating gate in NVMs^11^. Although some studies have investigated the integration of solution-processed NPs into OTFDs to mimic the charge-trapping mechanisms used in nanoscale solid-state devices that employ NPs, the challenge arises due to incompatibility with traditional CMOS processes^14–22^. Additionally, no previous study has investigated single CP-NP-based memory. Herein, we report a nano-memory Metal–Insulator–Semiconductor (MIS) structure that utilizes an acetylene-linked Pyrene–Isoindigo (**PI**) 2D-CMP NPs embedded in high-κ ALD oxide on bulk Ge. Conductive atomic force microscopy (cAFM) provides insight into the structure’s charge-trapping properties using local IV characterization and current mapping. The developed **PI**-based structure has the potential to be applied in next-generation flexible electronics and memory devices.

## Experimental method

### PI 2D-CMP synthesis and characterization

**Figure 1:**
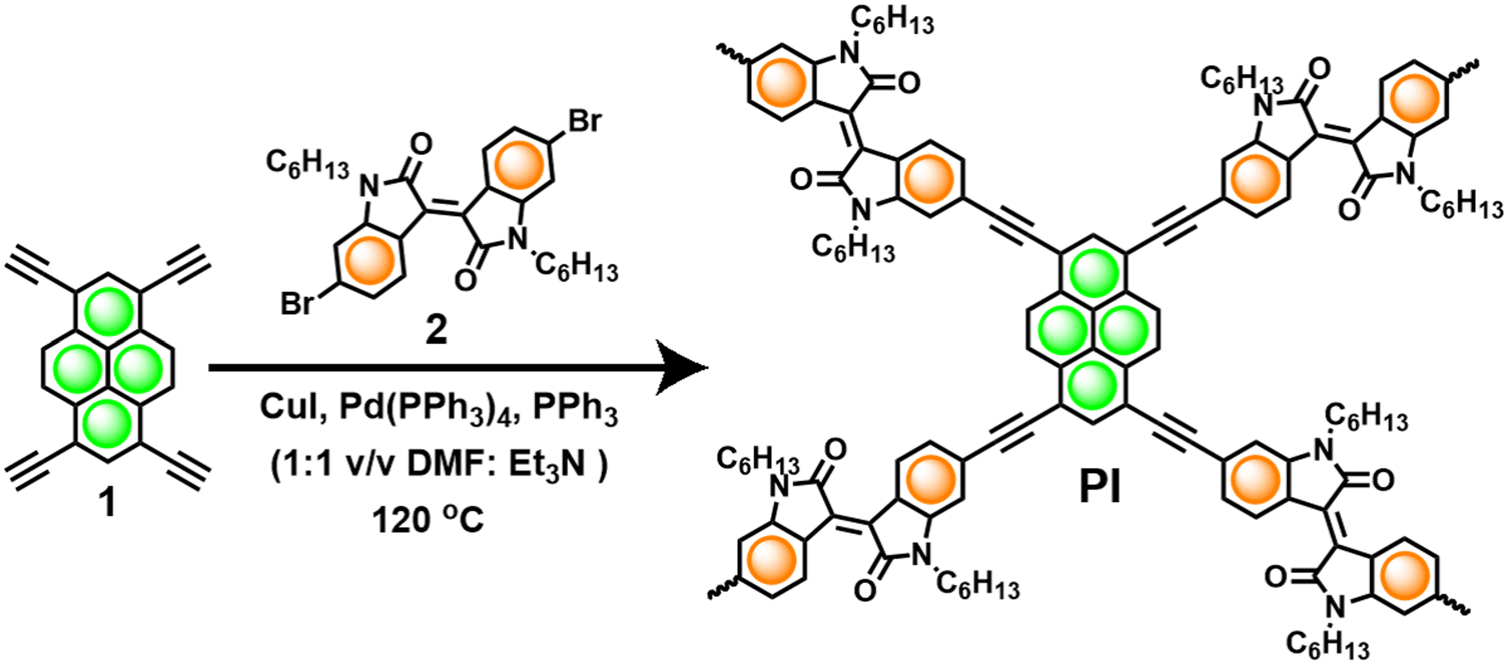
Synthetic approach for the rationally designed Pyrene-Isoindigo 2D-CMP (**PI**)^23^.

Donor–acceptor-based 2D CMP polymer based on pyrene and isoindigo (Fig. 1) was synthesized by following the reported procedure^23^. The synthesized polymer was well-characterized before testing it for further applications. In short, the Fourier-transform infrared (FT-IR) spectrum of **PI** showed the absence of ≡C–H and C–Br stretching bands corresponding to the monomers and the presence of alkyne –C≡C– stretching band to confirm the network formation (Fig. S1). Furthermore, molecular-level characterization is realized by solid-state NMR analysis (Fig. S2) whereas bulk microscopic characterization is achieved by scanning electron microscopy (Fig. S3). To understand the 2D nature of the polymer, Transmission Electron Microscopy (TEM) characterization is used. The images (Fig. S4) indicate the formation of stacked thin 2D-sheets to confirm the dimensionality of our material. The observation is further endorsed by the structural and morphological characterization described in our previous report^23^. The stacking of 2D-layers is governed by the strong van der Waals interaction originating from the *N*-hexyl chain and the large local dipole of isoindigo units and a strong π–π stacking interactions of pyrene units.

### MIS fabrication

To start the fabrication process, a germanium (Ge) (100) wafer is used as the starting material. The wafer is first cut into 1 cm × 1 cm pieces and then subjected to sonication in de-ionized (DI) water for 15 min to etch Ge native oxide. The pieces are then rinsed sequentially in acetone, isopropanol (IPA), and DI water. A 2 nm of Al_2_O_3_ is then grown on the prepared pieces using an Oxford FlexAl Plasma Atomic Layer Deposition (ALD) system. The precursor used for the deposition of the high-k oxide was trimethyl-aluminium, and the co-reactant employed was a remote O_2_ plasma. Growth is conducted at 200 °C and an operating pressure of 200 mTorr, ending up with a 0.12 nm/cycle growth rate. The TMAl pulsing and purge times are 0.25–4 s, respectively.

**Figure 2:**
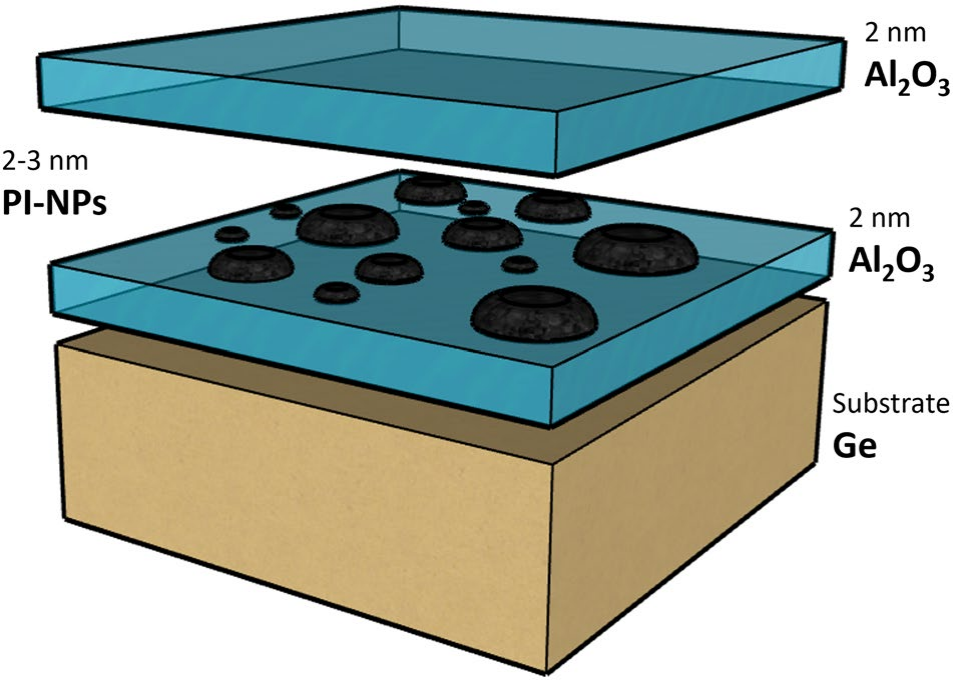
A schematic of the fabricated MIS structure with the embedded **PI**–NPs, showing the process flow.

The synthesized **PI**–NPs that have undergone the exfoliation process were suspended in IPA and stabilized with a concentration of 1 mg/mL. The solution was then immediately spin-coated onto the Al_2_O_3_ blocking oxide layer (BO). A volume of 20 µL was dropped on each sample while spinning at 500 RPM, raised to 1000 RPM for 45 s. To prevent any unwanted contamination from forming traps between the two alumina layers, an additional Al_2_O_3_ tunneling oxide (TO) was deposited immediately with a thickness of 2 nm, capping the **PI**–NPs. The thickness and roughness of the ALD films were verified using ellipsometry and AFM. Figure 2 illustrates the schematic of the fabricated structure. The **PI**–NPs size and dispersion along with the alumina BO/TO thicknesses were optimized  and engineered for their charge trap properties.

### MIS characterization

**Figure 3:**
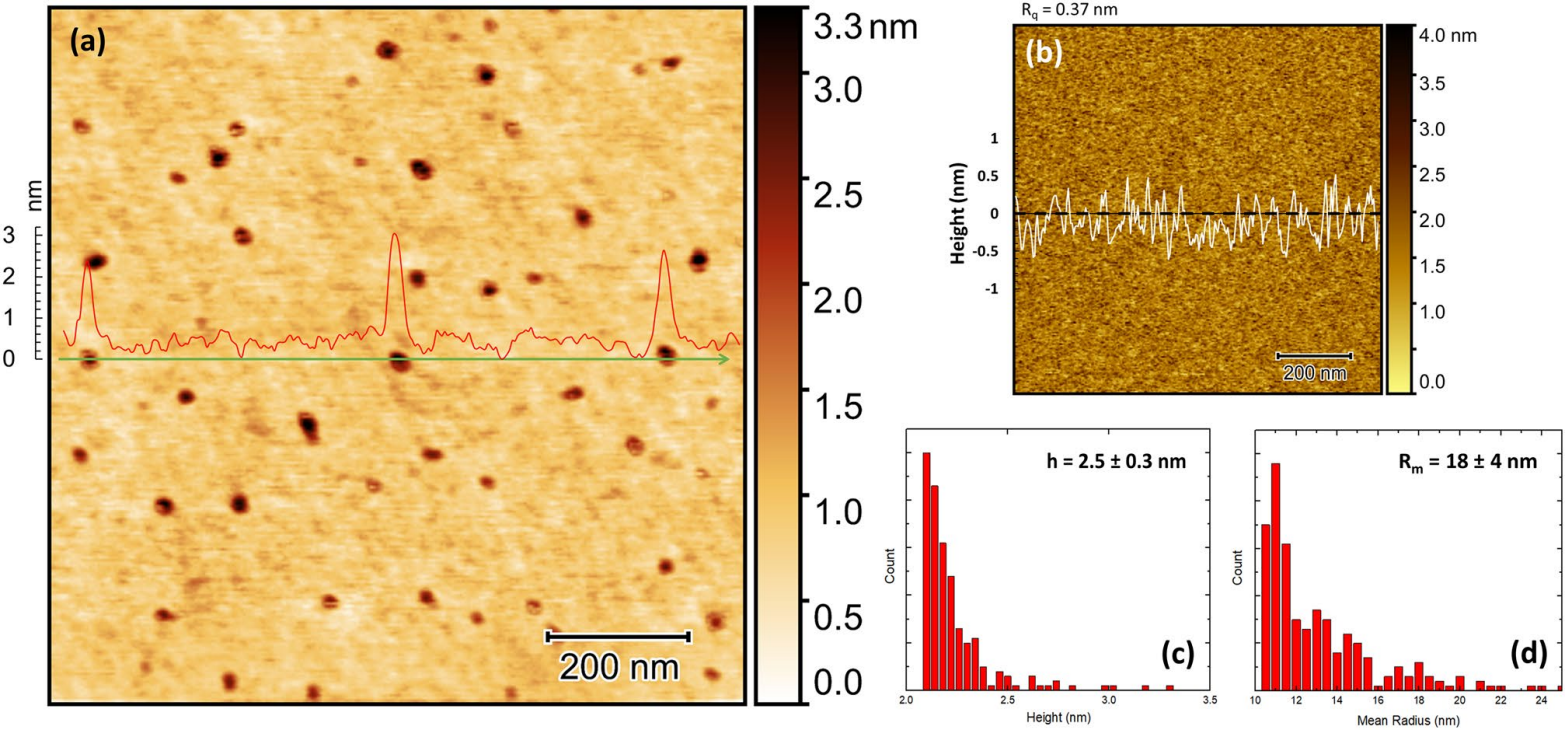
(**a**) Topography AFM scans in AC mode of the surface with the spin-coated **PI**–NPs on top of the Al_2_O_3_ BO along with the associated. (**b**) AFM scan of the BO before spin-coating and the RMS roughness. (**c**) Height and (**d**) mean radius distribution of **PI**–NPs.

The spatial characterization of well-separated **PI** NPs and their size were determined using AFM for samples without an Al_2_O_3_ tunneling oxide layer. Figure 3 depicts the AFM topographic image of the **PI**–NPs dispersion on top of the Al_2_O_3_ and their mean radius, respectively. The scans reveal that the exfoliated particles have a density of 9 µm^−2^ and a mean grain size of 50 nm. The AFM scans reveal that the exfoliated particles are dispersed and well separated with a mean grain radius ranging from ~ 10.5 to 25 nm along with a thickness ranging from ~ 2.1 to 3.3 nm.

**Figure 4:**
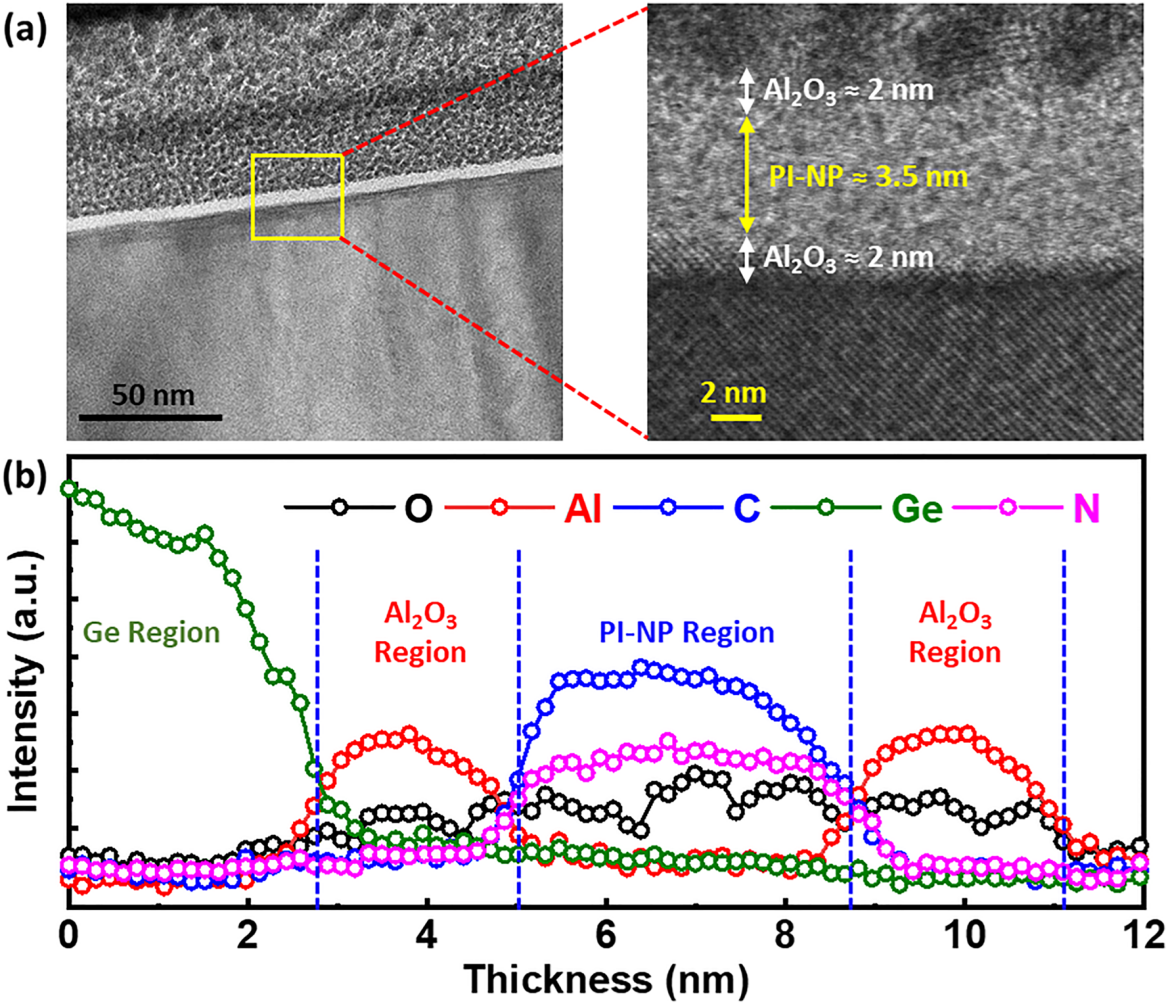
(**a**) Cross-sectional transmission electron microscopy (XTEM) image of the Pt-coated structure, showing the fabricated MIS stack with the embedded **PI**–NPs. (**b**) EELS compositional mapping of the stack.

Figure 4a shows cross-sectional transmission electron microscopy (XTEM) image of the fabricated MIS stack with the embedded **PI**–NPs. An obvious sandwich of the Al_2_O_3_ TO (2 nm), **PI**–NPs (3.5 nm), and Al_2_O_3_ BO (2 nm) can be observed (Fig. 4b). The TEM images are conducted using ThemisZ S/TEM operated at 300 kV. The samples were prepared using Helios G4 PFIB, a dual beam scanning electron microscope (SEM) that is also equipped with a focused ion beam (FIB) and an Omniprobe. The sample compositional and relative thickness mapping were conducted using a oxford energy-dispersive X-ray spectrometer (EDS) as shown in Fig. 4b.

**Figure 5:**
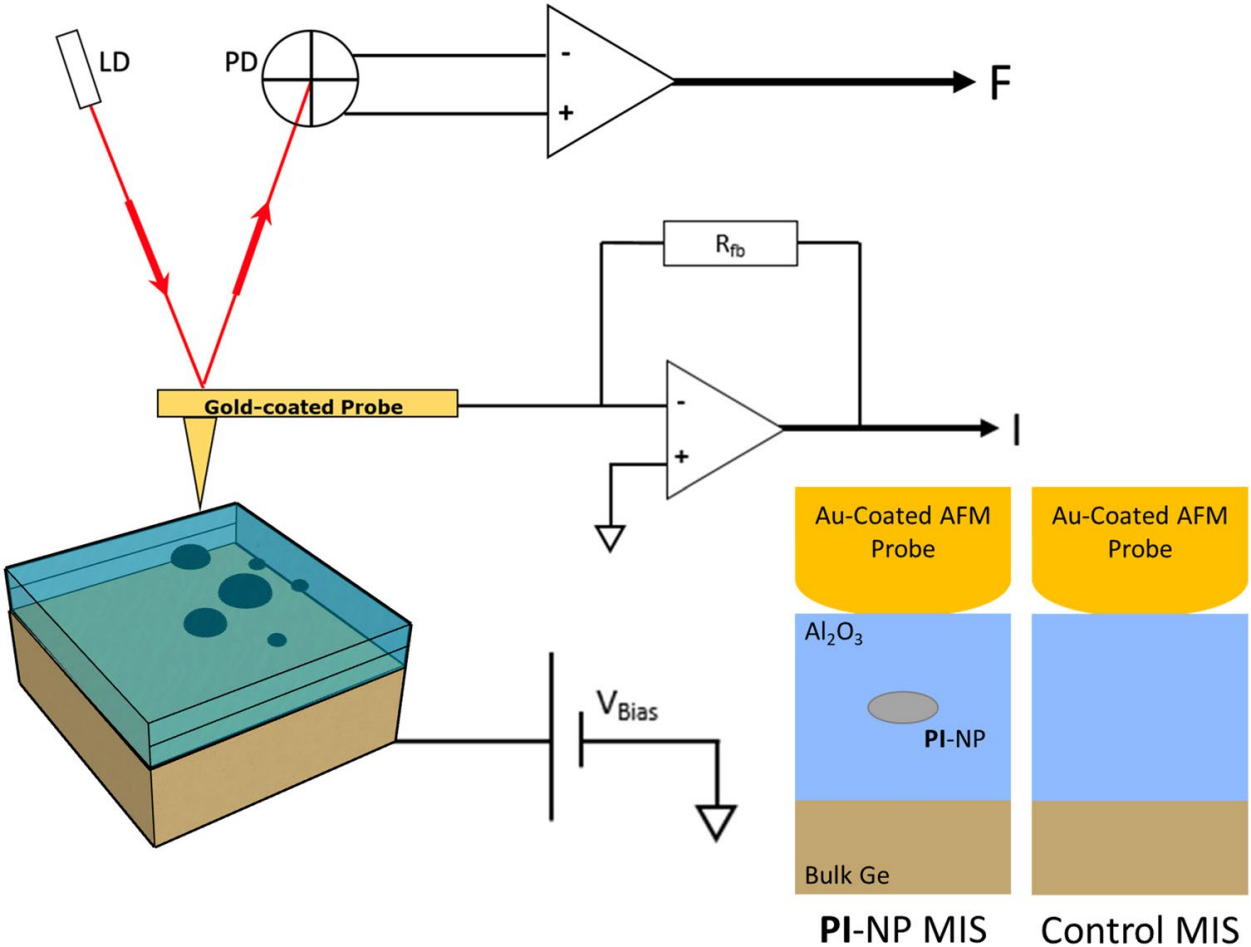
(**a**) The circuit of surface mapping and electrical probing of a single **PI**–NP using conductive-AFM. Inset shows a cross-section view of the probed MIS stack with and without the **PI**–NP.

Asylum Research MFP-3D AFM equipped with the (908.036) probe holder is used for both topography and electrical measurements. The probe holder allows current ranges of 1pA to 20nA, and voltage sweeps of ± 10V with a sensitivity of 2nA/V. Imaging in both contact and tapping mode was conducted with a (PPP-NCSTAu) gold-coated silicon probe. A diamond pen was used to scratch the back side of the samples and silver paste was applied before placing the sample on a metallic disk, assuring ohmic contact. The conductive probe was kept at a virtual ground while the Ge substrate’s back was biased. Once an isolated particle was located using topography and current mapping, the probe was situated on it, and potential sweep sequences are applied (refer to Fig. 5).

## Results

**Figure 6:**
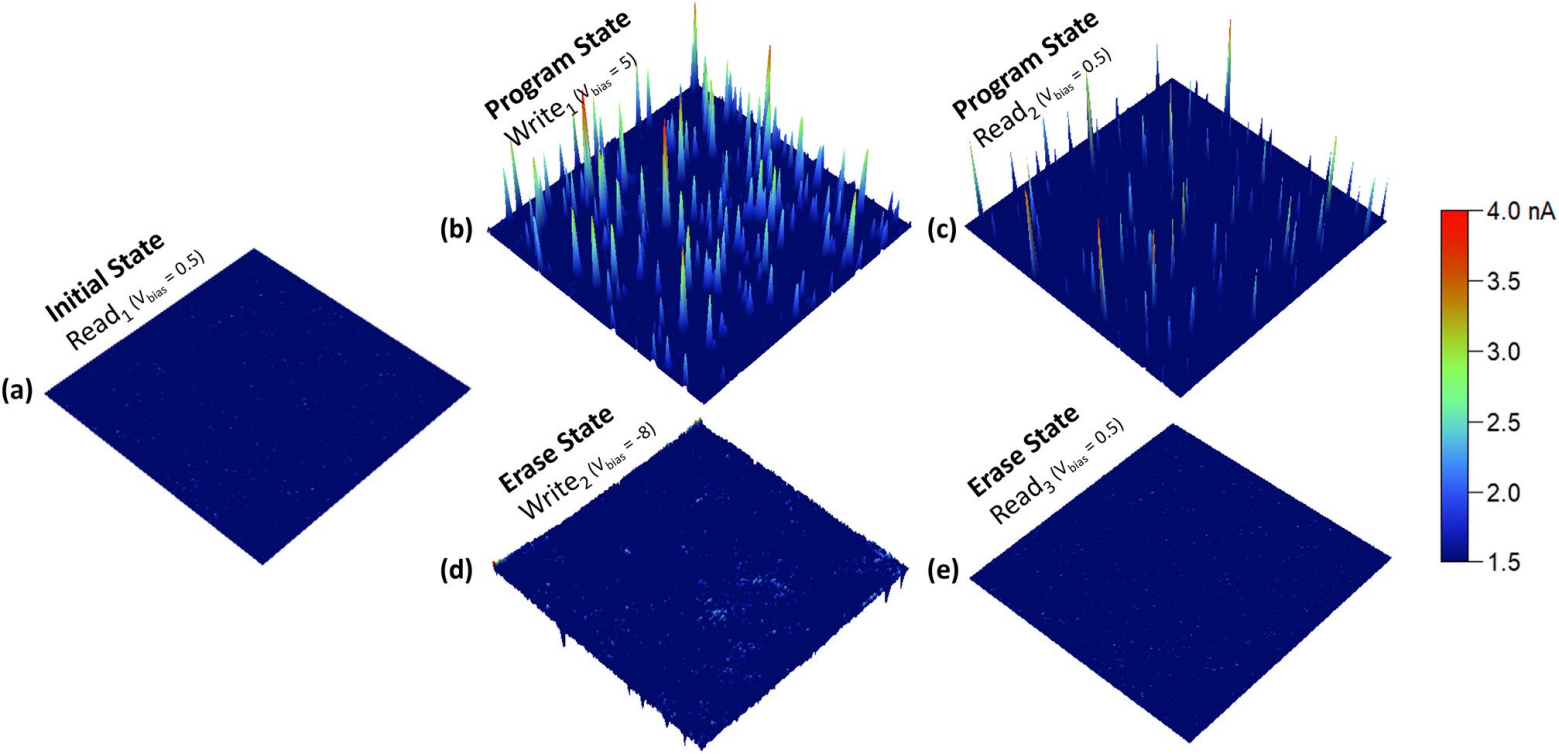
2.5 × 2.5 µm^2^ current maps of the (**a**) initial, (**b**) write, (**c**) read, (**d**) erase and (**e**) read scans.

We conducted a series of current scans using the cAFM, operating in contact mode, with dimensions of 2.5 × 2.5 µm^2^. The scans were carried out as follows: a read scan was performed at 0.5 V (Fig. 6a), a write scan was done at 5 V (Fig. 6b), followed by another read scan at 0.5 V (Fig. 6c), an erase scan was executed at − 8 V (Fig. 6d), and lastly, a final read scan was carried out at 0.5 V (Fig. 6e). It was observed that when the pristine device was subjected to the low-read voltage (0.5 V), no currents were detected. However, under a higher write voltage (5 V), current readings can be detected. The current readings, which were produced by the write scan, were also visible during the subsequent low-read voltage scan (0.5 V). These same currents were seldom visible after any erase scan (− 8 V) or during the succeeding read scan (0.5 V), as depicted in Fig. 6.

**Figure 7:**
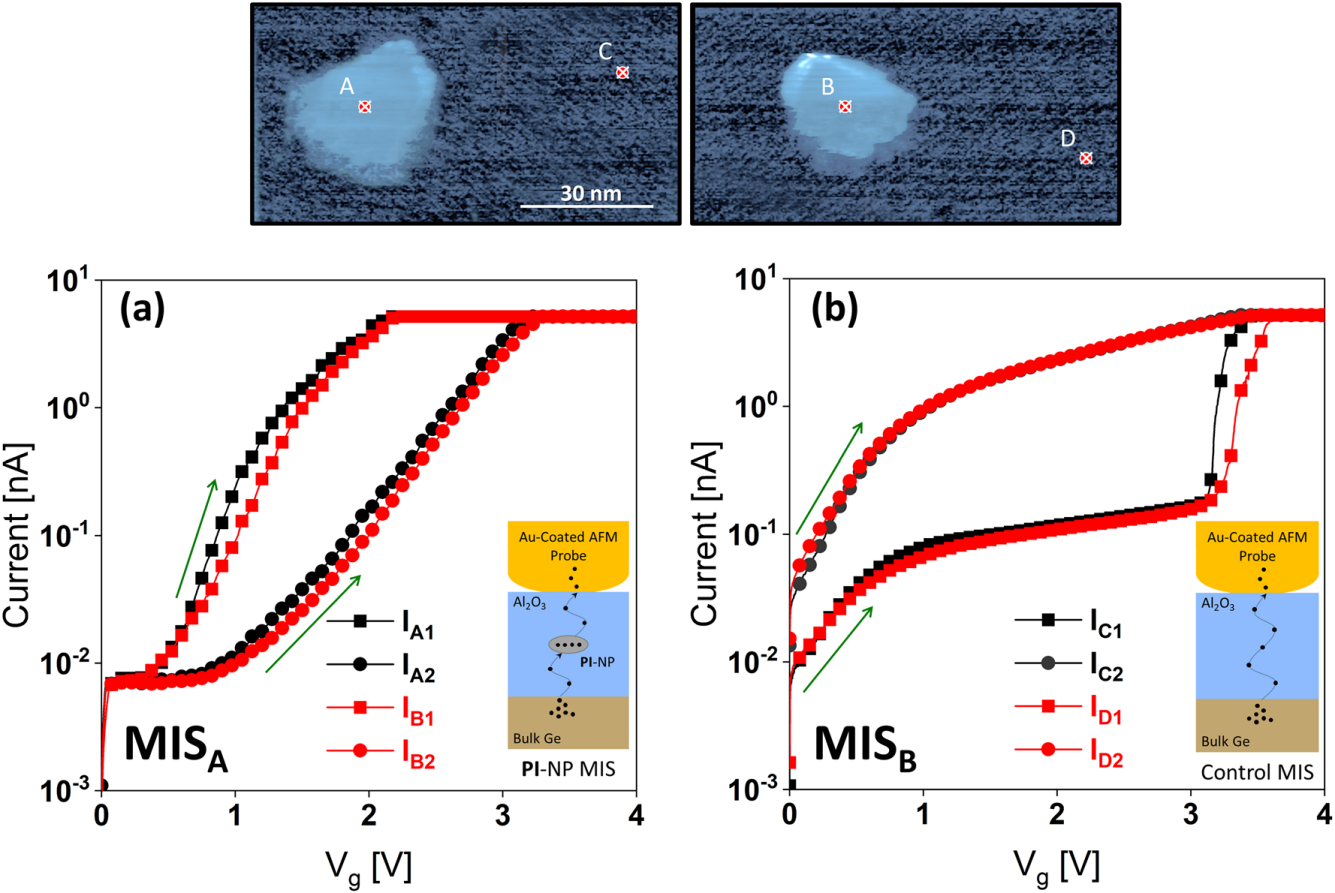
I-V characteristics for two successive sweeps at points A and B of the (**a**) MIS_A_ stack, as well as for points C and D of the (**b**) MIS_B_ stack.

We also demonstrate the charge storage component of our **PI**–NPs in MIS devices by conducting electrical measurements in the form of voltage sweeps and collecting the current response at predetermined locations. For clarification, MIS_A_ is used to refer to the **PI**–NPs-based MIS structure of Au-Tip/Al_2_O_3_/**PI**–NPs/Al_2_O_3_/Ge, while MIS_B_ refers to the control MIS structure of Au-Tip/Al_2_O_3_/Ge. Our findings, depicted in Fig. 7a and b, reveal distinct behaviors for the two stacks. Specifically, we observed current–voltage (I–V) characteristics for points A and B of the MIS_A_ stack, as well as for points C and D of the MIS_B_ stack. To ensure the reproducibility of the electrical behavior of MIS_A_ and MIS_B_, we performed I–V characteristic measurements at various locations, which demonstrated similar behavior.

By referring to Fig. 7a, it can be noted that the turn-on voltage of MIS_A_ at NPs A and B is much lower (approximately 0.9 V) when compared to that of MIS_B_ at oxides C and D (approximately 3.2 V) in Fig. 7b. At points C and D, in the absence of NPs, there is a distinct change in the I–V response at low bias voltages, where no current is detected as in the MIS_A_ stack. Nevertheless, as the bias voltage increases, a sudden rise in current response can be observed, signifying a breakdown in the oxide film. This is indicative of the creation of a conducting filament throughout the oxide layers under the first potential sweep. Nonetheless, the setting and resetting mechanisms of channels within dielectrics, such as Al_2_O_3,_ have been previously studied and are not the emphasis of the present work^24,25^.

The current responses after the first voltage sweep are consistently smaller compared to that of the latter, regardless of the reading voltage. This reduction in current response throughout the subsequent sweeps occurs because of carrier injection and confinement within the NP during the first sweep. As illustrated in Fig. 7a, a voltage shift (∆*V*) around 1 V is obtained between the first and second sweep at 200 pA reading current for both NPs. The window size is sufficient to differentiate between the two on and off states and, as a result, demonstrates the memory effect. The electron charge trapping behavior is experimentally verified by the positive voltage shift (∆*V*) in the *I*–*V* characteristics conducted on the MIS device structure, therefore confirming that the 2D-CMP behaves as an *n*-type semiconductor.

In Fig. 7, during the initial voltage sweep, MIS_A_ is configured to a low state, leading to subsequently lower current responses, while MIS_B_ is set to a high state. MIS_B_ functions as a resistive switching memory by altering the resistance of Al_2_O_3_, whereas MIS_A_ operates as a charge trapping memory, trapping, and de-trapped charge carriers within the **PI**–NP. Charge trapping memory typically exhibits slower operational speeds and a smaller memory window when compared to resistive switching memory. However, it often exhibits higher endurance, improved retention along with seamless integration with conventional CMOS technology^26,27^.

**Figure 8:**
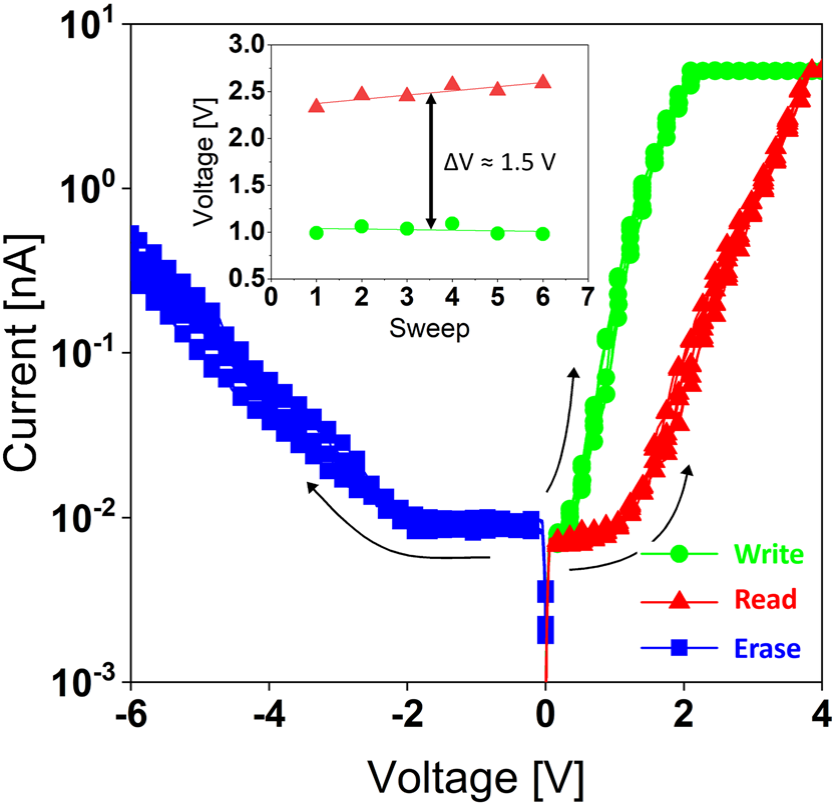
Six sets of successive write (green), read (red), erase (blue) IV sweeps. Inset highlights a memory window of 1.46 V at 200 pA.

For a more comprehensive investigation of the shift in the current response of the MIS_A_ stack throughout the initial and subsequent voltage sweeps, a series of individual potential scans can be used to read, write, and erase the charge stored in the **PI**–NP. These sweeps function as both charging (writing), charge detection (reading), and discharging (erasing) of the **PI**–NP. The entire process is depicted in Fig. 8, where a memory window (∆*V* = 1.46 V at 200 pA) is apparent between the initial and subsequent sweeps. Initially, no carriers are confined within the NP, so the first current response (W_1_), to a gate voltage sweep (*V*_g_) of 4 V, displays the charge transport and confinement within the NP (writing). Carriers tunnel across the Al_2_O_3_ TO only to get trapped in the NP due to the BO. On the other hand, the second response (R_1_) to the same gate sweep indicates a reduction in electric current due to the field screening introduced by the confined negative charge during the initial sweeps (reading). The lower current observed after the subsequent sweep further verifies the storage of charged carriers in the NP. The third response (E_1_) with a sweep of -6 V with opposite polarity is applied demonstrating the removal of stored charge (erasing) indicating it was sufficient to eject all confined charge. Charge ejection is verified with a follow-up potential scan of 5 V as seen in the fourth response (W_2_), where it almost coincides with those of the initial response. Further voltage sweeps overlap with the initial response (W_1_), signaling the reproducibility of the memory effect, as indicated by the six (Write/Read/Erase) sets of sweeps in Fig. 8. The I–Vs behavior presented illustrates a higher current flow under positive bias, which can be attributed to the improved tunneling due to the electric field enhancement at the nanoscale conductive probe and NP, narrowing the barrier width at the interface.

As illustrated by Figs. 2, 4 and 5 nm thick Al_2_O_3_ layers serve as an effective TO for the injected charge in the **PI**–NP sandwich, and act as an efficient BO for confining the charge. Nonetheless, there is a small contribution of Al_2_O_3_ to charge trapping in the system. Given the reported interface trap density within ultrathin Al_2_O_3_ thin films, which is approximately 10^11^–10^12^ cm^−2^^28^.

**Figure 9:**
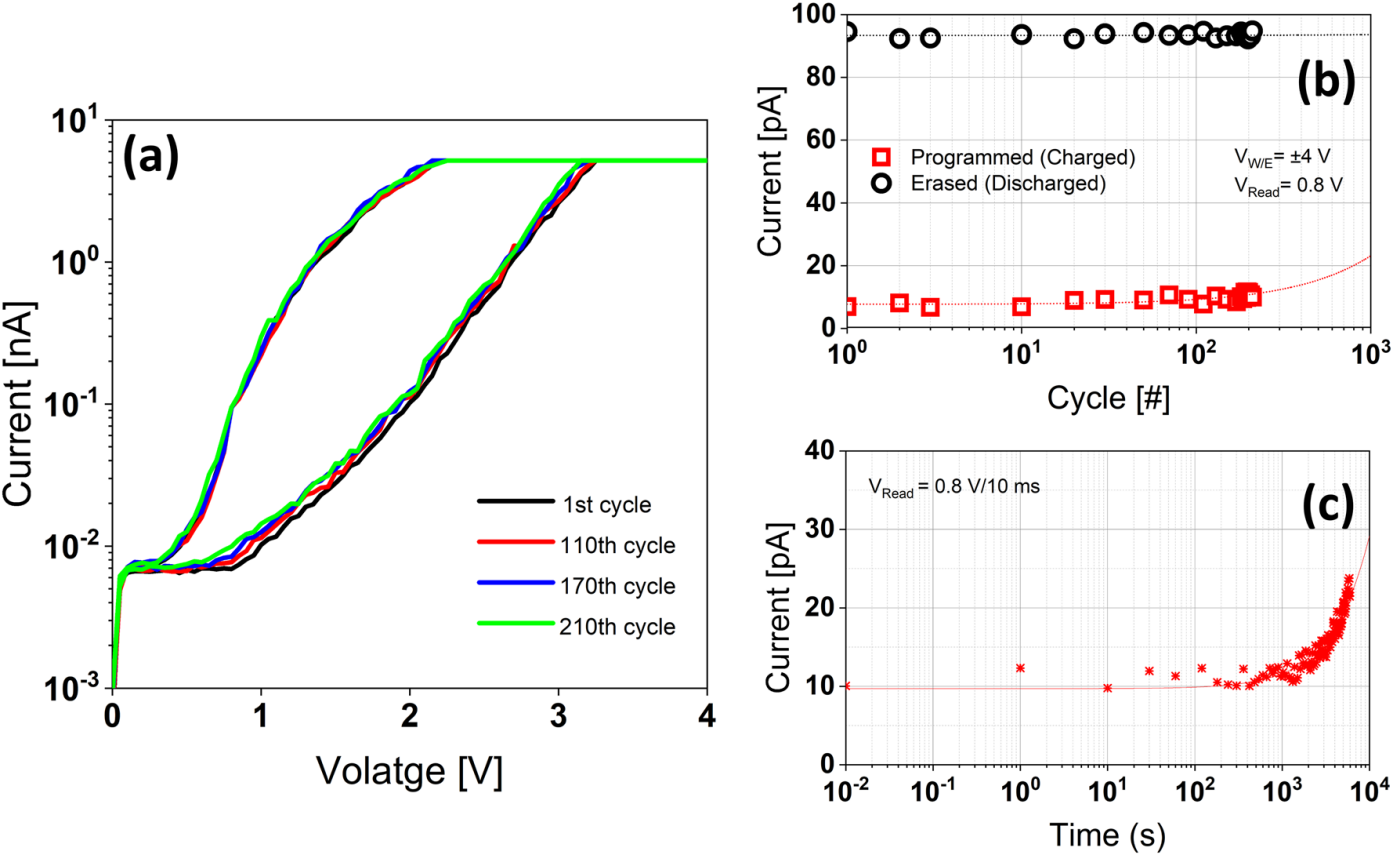
(**a**) IV sweeps of MIS_A_ structure throughout the endurance test at V_W/E_ =  ± 4 V after 1, 110, 170 and 210 cycles. (**b**) Endurance characteristics for 210 W/E cycles extracted at V_Read_ = 0.8 V from IV sweeps. (**c**) Retention characteristics up to 10^4^ s at V_W/E_ =  ± 4 V and V_Read_ = 0.8 V.

While Fig. 8 only illustrates the initial six write/erase (W/E) cycles, we also assessed the endurance characteristics of the MIS_A_ stack up to 210 cycles. The endurance measurement starts with the application of a W/E voltage pulse at ± 4 V for a given cycle number, followed by voltage sweeps from 0 to 4 V. Subsequently, Fig. 9a displays the *I-V* characteristics of the MIS_A_ stack throughout the endurance test, following 1, 110, 170, and 210 cycles. Additionally, Fig. 9b presents the current response recorded after a given cycle number, measured at 0.8 V, and extracted from 22 *I–V* sweeps. Even when extrapolated to 10^3^ W/E cycles, there is only a marginal 25% decrease in the memory on/off ratio. It is worth noting that while the reduction in discharged current remains minimal, the programmed current exhibits more pronounced increases as each erase process removes less charge compared to the write process therefore blocking less carriers, aligning with the asymmetrical nature of the IV curves. Additionally, it is plausible that local defect sites within the oxides facilitate increased electron tunneling, particularly under a high number of cycles.

We also evaluated the retention characteristics of the MIS_A_ device, as depicted in Fig. 9c. To monitor charge leakage, the retention measurement starts with a write voltage pulse at 4 V/100 ms, followed by the measurement of the current response using a read voltage pulse of 0.8 V/10 ms after a specified retention time interval, employing a custom pulse train. After ~ 1300 s, there is an observable increase in current response attributed to the reduction in trapped electric charge and, consequently, the diminishing shielding effect. Nonetheless, even after ~ 6000 s, the stored charges retained roughly 85% of their initial pristine value. The device exhibited good charge storage retention properties, characterized by a high on/off ratio memory, with a retention duration exceeding 10^4^ s.

**Figure 10:**
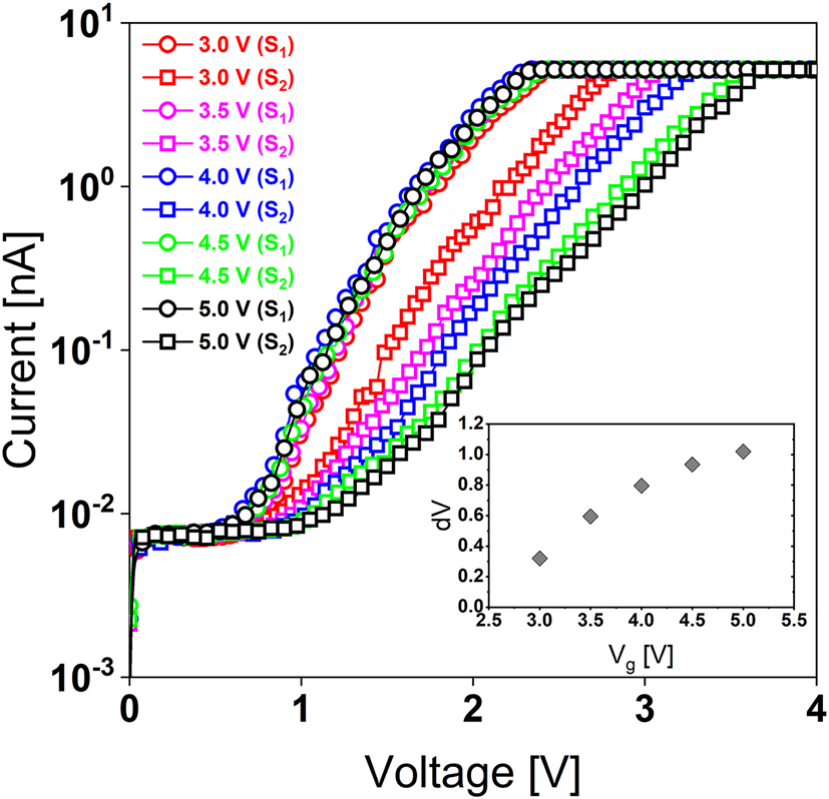
IV sweeps at different voltage biases. Inset shows the memory window vs bias voltage.

Figure 10 illustrates various write/read voltage biases when utilized to execute similar IV sweeps on a different **PI**–NP. By applying two successive gate voltage sweeps (*V*_*g*_) of 3, 3.5, 4, 4.5, and 5 V, resulting in a broad memory window up to (∆*V* = 1 V) at (*V*_g_ = 5 V at 200 pA).

While cAFM is a powerful tool to investigate the local electrical properties of materials at the nanoscale, it does have its limitations when it comes to current–voltage I-V and ultra-fast pulsed I-V characterization and lacks the capability for capacitance–voltage (C–V) measurements. The technique provides qualitative data about the electrical properties of the sample, but getting accurate quantitative data can be challenging due to the complexities of the electrical contact between the AFM tip and the sample. During measurements, the elevated local electric fields at the AFM tip can introduce additional stress to the device in addition to rapid and sustained current measurements can lead to rapid degradation of the AFM tip. Moreover, the cAFM technique exhibits a low signal-to-noise ratio, especially at low conductivities, and faces limitations in lateral resolution due to the dispersion of current within the material.

Previous work utilizing cAFM in similar memory structures usually employ resistive switching^29–31^. Resistive switching provides a larger memory window but usually requires significantly higher operation voltages and suffers from endurance issues. The few studies making use of charge trapping are limited but show similar memory window, retention, and endurance to our structure^15,32, 33^.

## Theoretical analysis and discussion

To affirm the charge confinement and transport within the produced MIS structures, a theoretical analysis (TA) is developed. The conducted TA starts with the calculation of charge capacitance (*C*_q_) and trapped charge density of (*N*_t_) and is analyzed through the charge-trapping mechanism^34^. The unknown dielectric constant value of **PI** is assumed to range from 1 to 3 based on previous work on polymers with similar chemical structure^35^. The total injected charge (*Q*_t_) is related to the threshold voltage shift (∆*V*_th_) by the following equation:

1 $$Q_{t} = C_{q} \cdot \Delta V_{th}$$

**Figure 11:**
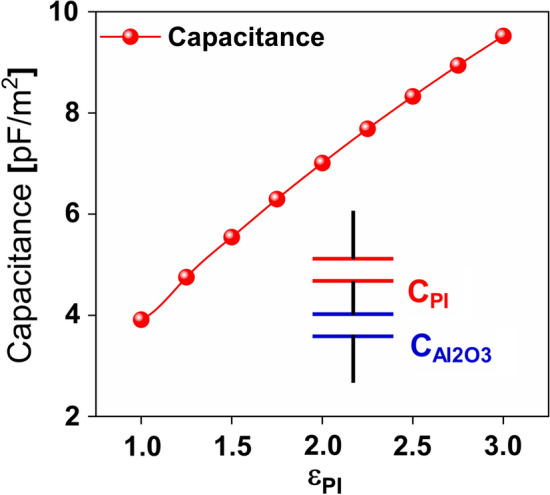
Capacitance at different dielectric constant values of **PI**.

The fabricated MIS structure (Fig. 7a) has a charge capacitance (*C*_q_) that can be expressed as two capacitors in series as depicted in Fig. 11:

2 $$\frac{1}{{C_{q} }} = \frac{1}{{C_{Al2O3} }} + \frac{1}{{C_{PI} }}$$

3 $$\frac{1}{{C_{q} }} = \frac{1}{{\frac{{\varepsilon_{0} \cdot \varepsilon_{Al2O3} }}{{t_{Al2O3} }}}} + \frac{1}{{\frac{{\varepsilon_{0} \cdot \varepsilon_{PI} }}{{t_{PI} }}}}$$

4 $$C_{q} = \frac{{\varepsilon_{0} \cdot \varepsilon_{Al2O3} \cdot \varepsilon_{PI} }}{{t_{Al2O3} \cdot \varepsilon_{PI} + t_{PI} \cdot \varepsilon_{Al2O3} }}$$

where *ε*_0_,* ε*_Al2O3_, and *ε*_PI_ are the dielectric constant of free space, Al_2_O_3_ and **PI**, respectively. While *t*_Al2O3_ and *t*_PI_ are the thicknesses of Al_2_O_3_ and **PI**–NP, respectively. Trapped charge density relies on various factors including the thickness of the oxide under the AFM probe, the linear dimension of the quantum well, and the layers’ relative permittivity. Therefore, the charge storage capacitance (*C*_q_) can be estimated for an *ε*_PI_ ranging from 1 to 3 as illustrated in Fig. 11 showing a maximum capacitance of 9.52 pF/m^2^ with an *ε*_PI_ of 3.0.

The trapped charge density (*N*_t_) can now be calculated from the charge capacitance (*C*_q_) for various *ε*_PI_ values using the following equation^36,37^:

5 $$N_{t} = \frac{{C_{q} \cdot \Delta V_{th} }}{q}$$

where ∆*V*_th_ is the change in threshold voltage of the on-and-off states of the memory and *q* is the elementary charge.

**Figure 12:**
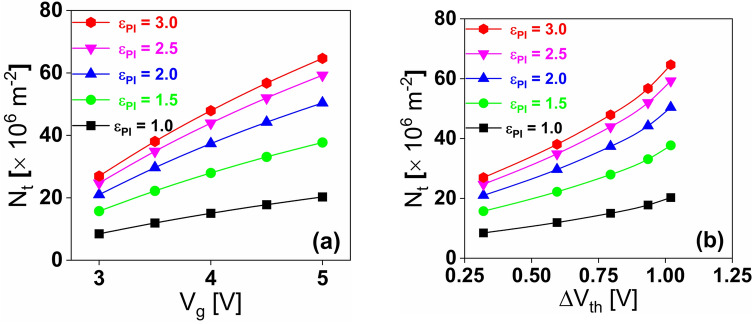
Variation in charge trap density with (**a**) applied gate voltage (*V*_g_) and (**b**) change in threshold voltage (Δ*V*_th_) for different dielectric constants of **PI**.

Figure 12a shows the trapped charge density (*N*_t_) for different ε_PI_ as a function of voltage shift (∆*V*_th_) which consent to the kinetics model of the charge transport where ∆*V*_th_ and *N*_t_ are linearly proportional. The same is reflected in Fig. 12b with a gate voltage (*V*_g_) variation.

Finally, the electric field across the tunneling oxide is calculated to investigate the charge emission mechanism. The electric field across the device is extracted by applying Gauss’s law to simple MIS devices^38^. The electric field across the tunneling oxide (*E*) is:

6 $$E = \frac{{V_{g} }}{{t_{Al2O3} + t_{PI} \left( {\frac{{\varepsilon_{Al2O3} }}{{\varepsilon_{PI} }}} \right) + t_{Al2O3} \left( {\frac{{\varepsilon_{Al2O3} }}{{\varepsilon_{Al2O3} }}} \right)}} + \frac{{N_{t} }}{{\varepsilon_{Al2O3} + \varepsilon_{PI} \left( {\frac{{t_{Al2O3} }}{{t_{PI} }}} \right) + \varepsilon_{Al2O3} \left( {\frac{{t_{Al2O3} }}{{t_{Al2O3} }}} \right)}}$$

**Figure 13:**
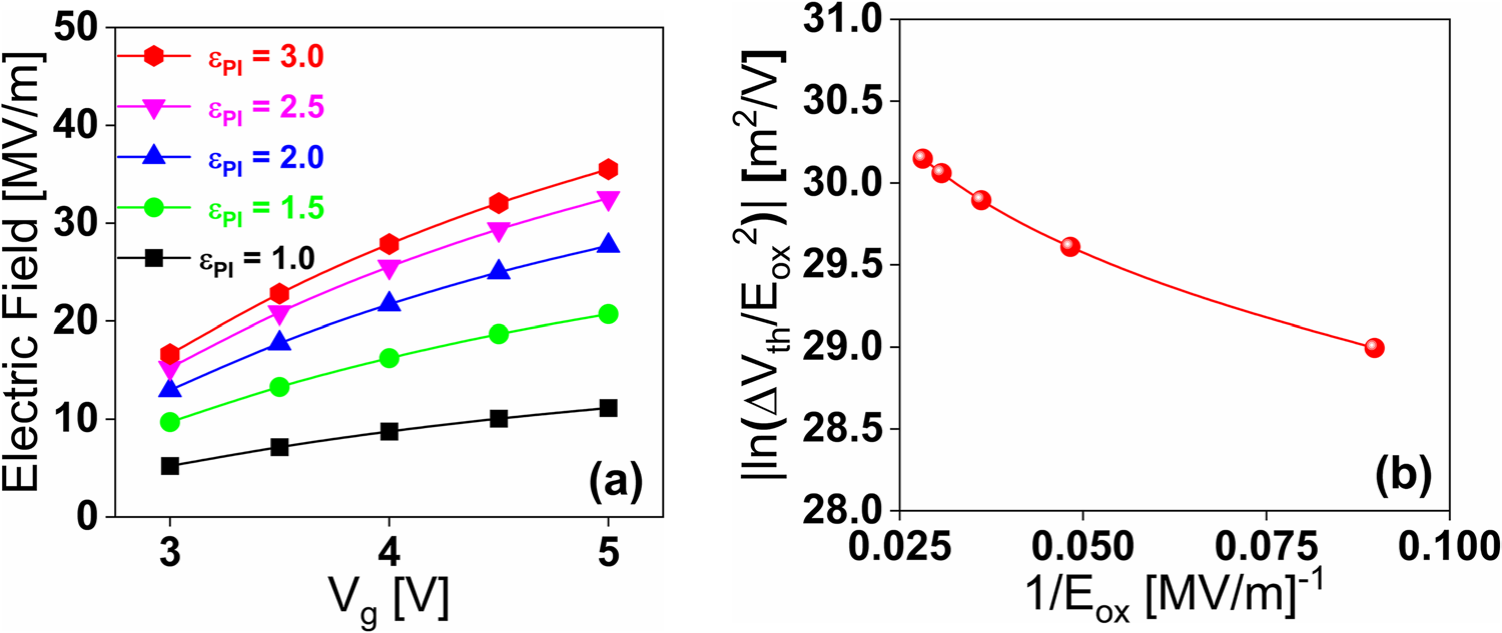
(**a**) Variation in Electric field across the tunneling oxide with gate voltage for the different dielectric constant of **PI**. (**b**) Variation in natural log of (Δ*V*_th_/E) with (1/E) across the tunneling oxide for *ε*_PI_ = 3.0.

The electric field (*E*) under various gate voltages (*V*_*G*_) is shown in Fig. 13, which reflects the enhancement of the electric field across the tunnel oxide due to the nanoscale size of the AFM probe. The initially higher electric field across the tunnel oxide conveys that the charge-trapping mechanism is based on Fowler–Nordhiem (F–N) tunnelling. This can be confirmed by plotting the natural log of (∆*V*_th_) divided by the squared inverse of the electric field (*E*) in Fig. 13b for *ε*_PI_ of 3.0. The linear trend confirms that the dominant charge emission mechanism is based on F-N tunneling.

**Figure 14:**
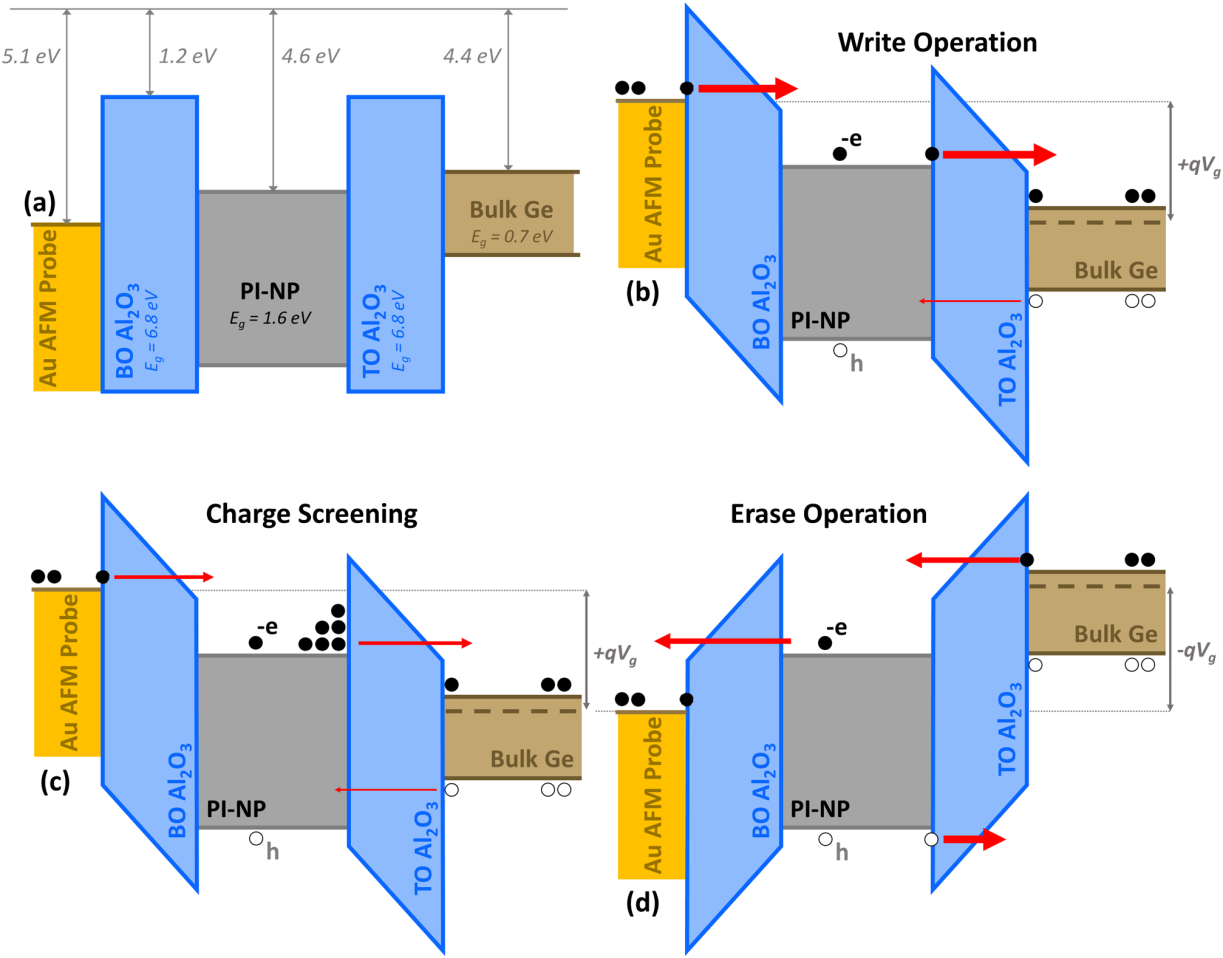
The energy band diagram for (**a**) flat band state, (**b**) charge injection during write operation, (**c**) charge screening, and (**d**) charge ejection during erase operation within the embedded **PI**–NP when probed using a conductive AFM Tip. The varying thickness of the red arrows symbolizes the magnitude of tunneling current.

Figure 14 demonstrates the F–N tunneling mechanism employing the energy band diagram of the MIS_A_ structure. During the *writing* operation shown in Fig. 14b, upon applying a sufficiently positive bias (V_g_) to the substrate, a very high electric field forms beneath the cAFM Au-coated tip, leading to an elevated Au-tip energy band. Electrons within the Au-tip acquire sufficient energy to be easily injected into the conduction band of **PI**–NP through the blocking oxide (BO) and become confined into states residing within the **PI** bandgap. Simultaneously a smaller number of the holes within the bulk can acquire adequate energy to directly tunnel from the valence band of Ge through the tunnel oxide (TO) and become confined also. This leads to the trapping of surplus of electrons within the **PI**–NP and interface states. Upon the application of subsequent positive bias as depicted in Fig. 14c, electrons encounter a shielding effect and are screened by the trapped charge, and hence, fewer electrons can tunnel causing a rightward shift in the memory's I–V characteristic. Conversely, during the *erase* operation as illustrated in Fig. 14d, a negative bias (− V_g_) is applied to the substrate causing an elevation of the Ge energy band. This promotes the tunneling of the stored electrons in the **PI**–NP back, whereas holes confined within the **PI**–NP can easily tunnel back into the bulk. The absence of trapped electrons results in the I-V characteristic of the memory shifting back to its initial state. This description aligns with our observations of the asymmetrical I-V curves under forward and reverse bias conditions which can be attributed to variations in conductance and valence band barrier heights at the interface.

Consequently, it can be inferred that a high density of trapping states exists in **PI**–NP with increased defect density, leading to an expanded memory window. However, achieving precise control over the defect density in our current device setup poses a significant challenge due to its 2D and porous nature. In future research endeavors, additional efforts will be required to enhance the precision of defect density manipulation in **PI**–NP. From an electron theory perspective, a low-lying LUMO, indicated by high electron affinity from Density Functional Theory (DFT) calculations on **PI**^23^, can serve as a charge-trapping center which in turn allow the capture of charged carriers from the Fermi energy level and tunneling from either the conductive probe or semiconductive substrate^39^.

## Conclusions

In summary, the fabricated MIS structure utilizes an acetylene-linked Pyrene-Isoindigo (**PI**) 2D-CMP NPs embedded in high-κ ALD oxide on bulk Ge. Conductive atomic force microscopy (cAFM) is used to provide insight into the structure’s charge-trapping properties using local IV characterization and current mapping. The device displays a wide memory window (∆V) up to 1.5 V at relatively small reading voltages along with reproducible on-and-off states. Furthermore, a theoretical analysis is developed to affirm the measured charge carriers’ transport and entrapment through and within the fabricated MIS structures. The developed **PI**-based structure has the potential to be applied in next-generation low-power high-density memory devices.

### Supplementary Information

Supplementary Figures.

## Data Availability

The data that support the findings of this work are available from the corresponding authors upon reasonable request.
