# Supplementary material for: Nano-scale charge trapping memory based on two-dimensional conjugated microporous polymer
Source: Sci Rep. 2023 Nov 1;13:18845. doi: 10.1038/s41598-023-44232-1 (PMC10620224; doi:10.1038/s41598-023-44232-1)
Supplement: Supplementary file 1 — Supplementary Figures. [file 41598_2023_44232_MOESM1_ESM.docx]

Supporting Information for

**Nano-Scale Charge Trapping Memory based on Two-Dimensional Conjugated Microporous Polymer**

Ayman Rezk^1^, Md. Hasan Raza Ansari^2^, Kayaramkodath Chandran Ranjeesh^3^, Safa Gaber^3^, Dayanand Kumar^2^, Areej Merhi^4^, Bilal R. Kaafarani^4^, Mohamed Ben Hassine^5^, Nazek El-Atab^2^, Dinesh Shetty^*3^, 6, Ammar Nayfeh^*1^

*^1^Department of Electrical Engineering and Computer Science, Khalifa University, Abu Dhabi, 127788, UAE. E-mail:* [*ammar.nayfeh@ku.ac.ae*](mailto:ammar.nayfeh@ku.ac.ae)*.*

*^2^Smart, Advanced Memory Devices and Applications (SAMA) Laboratory, Electrical and Computer Engineering Program, Computer Electrical Mathematical Science and Engineering Division, King Abdullah University of Science and Technology (KAUST), Thuwal 23955, Kingdom of Saudi Arabia*

*^3^Department of Chemistry, Khalifa University, PO Box 127788, Abu Dhabi, UAE.*

*E-mail:* [*dinesh.shetty@ku.ac.ae*](mailto:dinesh.shetty@ku.ac.ae)*.*

*^4^Department of Chemistry, American University of Beirut, Beirut 1107-2020, Lebanon.*

*^5^Electron Microscopy core Labs, King Abdullah University of Science and Technology (KAUST), Thuwal 23955, Kingdom of Saudi Arabia*

*^6^Advanced Materials Chemistry Center (AMCC), Khalifa University, PO Box 127788, Abu Dhabi, UAE.*

**Content**

| 1. Figures S1 to S4 | Page S2-S5 |
| --- | --- |

**3. Figures**

**Figure S1.** Fourier-transform infrared (FT-IR) spectra of **PI** and monomers.

**Figure S2.** Solid state ^13^Carbon cross-polarization magic-angle spinning nuclear magnetic resonance (^13^C CP-MAS NMR) spectra of **PI**

**Figure S3.** SEM images of **PI**.

**Figure S4:** TEM images of **PI**.
